# Evaluation of Environmental Contamination and Estimated Radiation Doses for the Return to Residents’ Homes in Kawauchi Village, Fukushima Prefecture

**DOI:** 10.1371/journal.pone.0045816

**Published:** 2012-09-26

**Authors:** Yasuyuki Taira, Naomi Hayashida, Hitoshi Yamaguchi, Shunichi Yamashita, Yuukou Endo, Noboru Takamura

**Affiliations:** 1 Department of Global Health, Medical and Welfare, Nagasaki University Graduate School of Biomedical Sciences, Nagasaki, Japan; 2 Department of Radiation Medical Science, Nagasaki University Graduate School of Biomedical Sciences, Nagasaki, Japan; 3 Department of Ecomaterials Science, Nagasaki University Graduate School of Engineering, Nagasaki, Japan; 4 Nagasaki Prefectural Institute for Environmental Research and Public Health, Omura, Japan; 5 Kawauchi Village Mayor, Kawauchi Municipal Government, Fukushima, Japan; University of California, San Francisco, United States of America

## Abstract

To evaluate the environmental contamination and radiation exposure dose rates due to artificial radionuclides in Kawauchi Village, Fukushima Prefecture, the restricted area within a 30-km radius from the Fukushima Dai-ichi Nuclear Power Plant (FNPP), the concentrations of artificial radionuclides in soil samples, tree needles, and mushrooms were analyzed by gamma spectrometry. Nine months have passed since samples were collected on December 19 and 20, 2011, 9 months after the FNPP accident, and the prevalent dose-forming artificial radionuclides from all samples were ^134^Cs and ^137^Cs. The estimated external effective doses from soil samples were 0.42–7.2 µSv/h (3.7–63.0 mSv/y) within the 20-km radius from FNPP and 0.0011–0.38 µSv/h (0.010–3.3 mSv/y) within the 20–30 km radius from FNPP. The present study revealed that current levels are sufficiently decreasing in Kawauchi Village, especially in areas within the 20- to 30-km radius from FNPP. Thus, residents may return their homes with long-term follow-up of the environmental monitoring and countermeasures such as decontamination and restrictions of the intake of foods for reducing unnecessary exposure. The case of Kawauchi Village will be the first model for the return to residents’ homes after the FNPP accident.

## Introduction

On March 11, 2011, a 9.0-magnitude earthquake (The Great East Japan Earthquake) struck the east coast near Iwate, Miyagi, and Fukushima Prefectures, Japan. The earthquake in combination with the tsunami caused extensive damage to the Fukushima Dai-ichi Nuclear Power Plant (FNPP) and a radioactive plume derived from Units 1, 2, 3, and 4 of FNPP was dispersed in the atmosphere. The total amount of radioactive materials released into the atmosphere from FNPP corresponds to Level 7 of the International Nuclear and Radiological Event Scale (INES) by the International Atomic Energy Agency (IAEA). Although this level corresponds to a major accident, the amount of radioactive materials released into the environment is estimated to be 10% [1.6×10^17^ Bq for ^131^I (half-life: 8.0 d) and 1.5×10^16^ Bq for ^137^Cs (30 y)] of the accident at the Chernobyl Nuclear Power Plant (CNPP, 1.8×10^18^ Bq for ^131^I and 8.5×10^16^ Bq for ^137^Cs), which was previously assessed at the same level [1].

Since March 12, 2011, a 20-km radius from FNPP has been stipulated as “a no-entry zone”. In the emergency zone outside a 20-km radius from FNPP, there are certain areas where radioactive materials emitted from the power station have accumulated as a result of climatic and geographical conditions and some of these areas have shown high levels of radionuclides.

However, levels of environmental radioactivity are decreasing due to the decay of short-lived radionuclides such as ^131^I, ^129m^Te (34 d), ^95^Nb (35 d), and ^136^Cs (13 d) and recovery efforts such as the remediation of contaminated soil. Six months after the FNPP accident, designated emergency evacuation preparation zones within the 20- to 30-km radius from FNPP were canceled [2]. Moreover, the Japanese government announced on December 16, 2011 that it had confirmed that the reactors of FNPP are stabilized and that the accident in the station had been terminated once the reactors were brought to a condition equivalent to “cold shutdown” [2]. On January 31, 2012, the head of Kawauchi Village, the restricted area within the 30-km radius from FNPP, declared that residents could safely return to their homes because radiation doses were found to be at comparatively low levels. However, the return is still not progressing smoothly around FNPP.

For residents to return to their homes, it is extremely important to evaluate environmental contamination and radiation exposure risks due to the FNPP accident for the radiation protection and public health of residents. Although some reports showed environmental contamination and estimated exposure doses during the initial phase of the accident, evaluation of the radiation health risks due to the return to an area such as Kawauchi Village has not reported [3]–[6]. Therefore, for the evaluation of current environmental contamination and contributory external and internal exposures, we analyzed the concentrations of artificial radionuclides in soil samples, tree needles, and mushrooms collected in residential areas of Kawauchi Village by gamma spectrometry. Furthermore, external and internal effective doses were calculated from these samples for the estimation of radiation exposure status.

**Figure 1:**
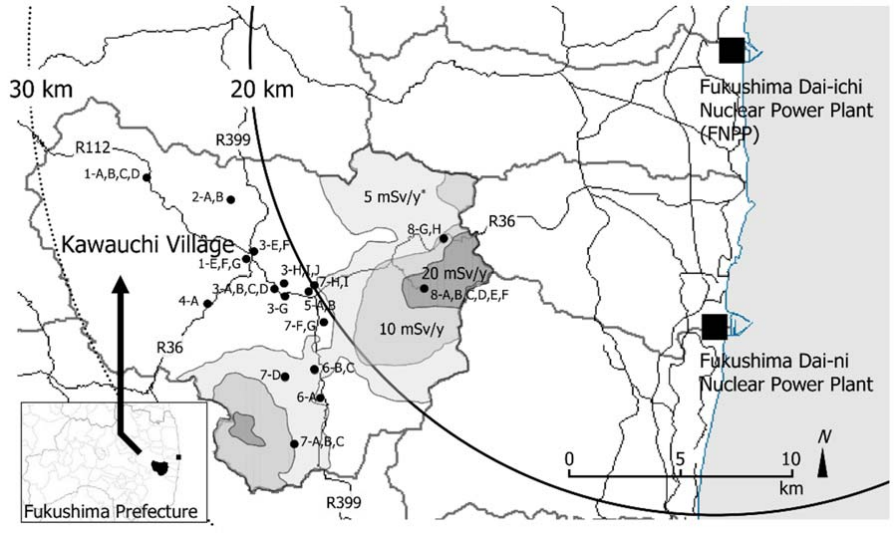
Location of sampling points, Kawauchi Village, Fukushima Prefecture. Closed circles (•) are sampling points. The asterisk indicates the estimated annual dose based on the survey from the accident to December 11, 2011 by MEXT (Available: http://radioactivity.mext.go.jp/en/. Accessed on August 6, 2012).

## Materials and Methods

### Sampling Places

Fukushima Dai-ichi Nuclear Power Plant is located on the east coast of Honshu Island, approximately 200 km northeast of Tokyo. Samples around FNPP were collected at the following sites in Japan: Kawauchi Village (the public office, N37° 20′, E140° 48′), 22.0 km southwest of FNPP (N37° 25′, E141° 02′); Fukushima City (the prefectural office, N37° 41′, E140° 28′), 57.8 km northwest of FNPP; and Tokyo (N35° 41′, E139° 46′), 223.2 km southwest of FNPP.

**Table 1:** Distribution of detected artificial radionuclides and ^40^K, and ^134^Cs: ^137^Cs ratios in surface soils collected at Kawauchi Village, Fukushima Prefecture.

Points	Distance fromFNPP (km)	Radionuclide concentration in Bq/kg-dry			Radionuclide ratio
		^134^Cs (2.1 y)^a^	^137^Cs (30 y)	^40^K (1.3×10^9^ y)	^134^Cs/^137^Cs
1-A	25.5	5915±35^b^ (30)^c^	7208±39 (24)	703±50 (74)	0.82
1-B	25.5	398±6.8 (10)	543±7.8 (9.3)	511±30 (49)	0.73
1-G	22.5	1554±13 (14)	2015±15 (12)	888±40 (42)	0.77
2-A	22.2	2393±24 (23)	2962±27 (17)	999±63 (85)	0.81
3-A	21.7	20.0±1.7 (3.3)	25.4±1.9 (3.4)	853±37 (42)	0.79
3-B	21.7	2637±17 (17)	3416±20 (13)	834±38 (42)	0.77
3-C	21.7	495±8.0 (8.2)	618±8.9 (7.1)	803±40 (49)	0.80
3-D	21.7	219±4.5 (4.8)	275±5.2 (4.5)	786±34 (36)	0.80
3-F	21.8	2822±23 (21)	3472±26 (16)	744±49 (69)	0.81
3-G	21.4	1628±14 (17)	2011±16 (16)	606±35 (49)	0.81
3-H	21.5	629±7.3 (7.3)	803±8.4 (5.7)	600±28 (29)	0.78
3-I	21.5	2394±17 (15)	3016±19 (12)	630±34 (43)	0.79
4-A	24.7	6867±56 (48)	3570±61 (37)	638±75 (152)	1.92
5-A	20.5	3574±24 (21)	4480±27 (17)	445±36 (56)	0.80
6-A	23.0	7191±28 (30)	9158±32 (28)	783±37 (51)	0.79
6-B	22.4	1374±14 (18)	1763±16 (18)	569±37 (60)	0.78
7-A	25.1	4363±28 (25)	5442±31 (19)	923±52 (66)	0.80
7-D	23.5	222±7.1 (11)	300±7.9 (10)	658±45 (68)	0.74
7-F	20.8	5141±28 (24)	6449±32 (18)	669±42 (63)	0.80
7-H	20.0	30628±60 (48)	37108±63 (35)	131±38 (114)	0.83
7-I	20.0	1809±13 (12)	2282±15 (9.3)	832±35 (39)	0.79
8-A	16.8	272366±202 (178)	340331±226 (151)	559±67 (181)	0.80
8-B	16.8	9275±35 (30)	11699±40 (23)	569±36 (53)	0.79
8-C	16.8	20453±60 (50)	25576±67 (34)	480±45 (94)	0.80
8-D	16.8	28308±82 (67)	35248±90 (50)	467±51 (113)	0.80
8-G	14.9	29480±82 (67)	36474±90 (52)	645±55 (106)	0.81
F-1	62.1	3018±23 (21)	3782±26 (16)	536±41 (62)	0.80
T-1	223	9.0±1.4 (3.5)	11.0±1.6 (3.8)	401±30 (42)	0.82

a half-life.

b error shows one sigma standard deviation from counting statistics.

c detection limit.

In Kawauchi Village, 42 samples were collected at public facilities such as schools, assembly halls, a medical office, and forests along major roads. Sampling areas ranged from N37°17′ to N37°22′ and E140°45′to E140°53′, and distances from FNPP were between 14.9 and 25.5 km (**Figure 1**).

**Table 2:** External effective doses from surface soils due to radiocesium and radiation doses by a gamma-ray survey meter in Kawauchi Village, Fukushima Prefecture.

Points	Distance from FNPP (km)	External effective dose		Air dose rate in µSv/h
		µSv/h	mSv/y	
1-A	25.5	0.12	1.0	0.50
1-B	25.5	0.022	0.19	0.40
1-G	22.5	0.074	0.65	0.46
2-A	22.2	0.043	0.38	0.60
3-A	21.7	0.0011	0.010	0.18
3-B	21.7	0.14	1.2	0.18
3-C	21.7	0.019	0.17	0.18
3-D	21.7	0.015	0.13	0.60
3-F	21.8	0.074	0.65	0.38
3-G	21.4	0.082	0.72	0.44
3-H	21.5	0.045	0.39	0.48
3-I	21.5	0.11	0.99	0.48
4-A	24.7	0.047	0.41	0.37
5-A	20.5	0.11	0.94	1.1
6-A	23.0	0.38	3.3	0.82
6-B	22.4	0.058	0.51	1.8
7-A	25.1	0.10	0.86	1.2
7-D	23.5	0.0049	0.043	1.4
7-F	20.8	0.17	1.5	0.80
7-H	20.0	0.10	0.83	1.3
7-I	20.0	0.11	1.0	1.0
8-A	16.8	7.2	63.0	>20
8-B	16.8	0.42	3.7	5.7
8-C	16.8	0.56	5.0	5.0
8-D	16.8	0.51	4.5	6.4
8-G	14.9	0.54	4.7	2.3
F-1	62.1	0.074	0.65	1.5
T-1	223	0.00032	0.0028	0.10

Air dose rates in all sampling places were monitored in air 1 m above the ground by a portable detector for the management of radiation exposure (PDR-201®, Hitachi-Aloka Medical, Ltd., Tokyo, Japan).

**Figure 2:**
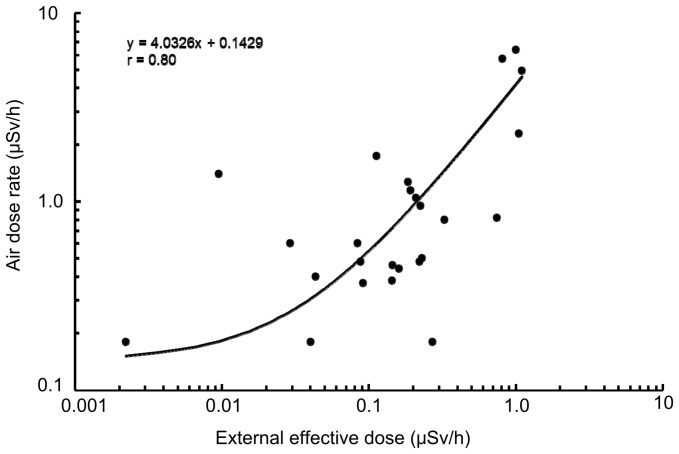
Relationship between estimated external effective doses from soils and air dose rates in Kawauchi Village. External effective doses were estimated using Eq. (1); *s* is the decrease in the coefficient by a shielding factor against exposure with gamma rays from a deposit at 1 m above ground (0.7 under the condition of usual land (IAEA-TECDOC-1162)). Air dose rates were monitored by a portable detector in sampling places (PDR-201®, Hitachi-Aloka Medical, Ltd., Tokyo, Japan). Excluded 8-A by uncertainly data of air dose rate.

**Figure 3:**
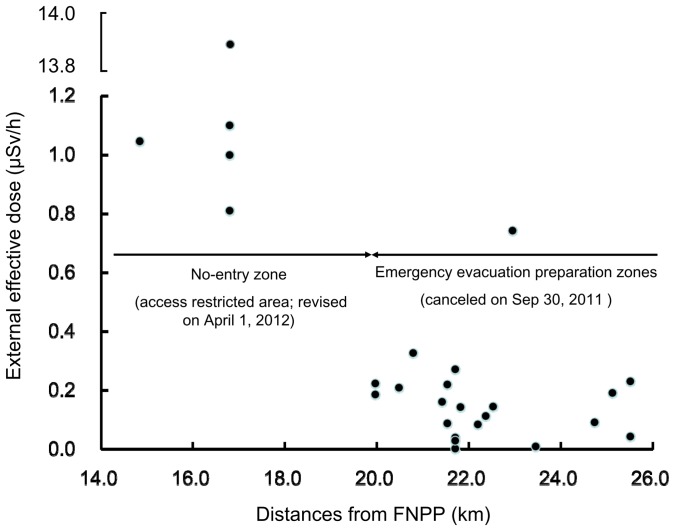
Relationship between distances from FNPP and effective doses from soil samples. External effective doses were estimated using Eq. (1) as in Figure 2.

### Measurement of Radionuclides

For the evaluation of external radiation exposure, 26 samples of undisturbed surface soil (0–5 cm) were collected at Kawauchi Village on December 19 and 20, 2011, and samples of surface soil were collected at Fukushima City on December 20, 2011 and at Tokyo on December 20, 2011 as control samples. Soil sampling was carried out by a core sampling technique at all sampling places. The size of the soil samples was 18.2 cm^2^ (a diameter of 4.8 cm) and the density of the surface soil layer ranged from 0.3 to 1.7 g/cm^3^-dry. At the same time, 13 samples of pine and cedar tree needles (fallen leaves) were collected at Kawauchi Village on December 19 and 20, 2011.

**Table 3:** Distribution of detected artificial radionuclides and ^40^K, and ^134^Cs: ^137^Cs ratios in needles collected at Kawauchi Village, Fukushima Prefecture.

Points	Distance fromFNPP (km)	Radionuclide concentration in Bq/kg-dry			Radionuclide ratio
		^134^Cs (2.1 y)^a^	^137^Cs (30 y)	^40^K (1.3×10^9^ y)	^134^Cs/^137^Cs
1-C	25.5	2880±27^b^ (23)^c^	3576±31 (18)	139±30 (78)	0.81
2-B	22.2	1159±14 (12)	1484±16 (10)	76.4±19 (51)	0.78
3-E	21.8	1737±19 (16)	2168±21 (13)	67.8±22 (65)	0.80
3-J	21.5	3842±18 (14)	4948±21 (12)	72.1±14 (38)	0.78
5-B	20.5	1796±18 (15)	2308±20 (12)	78.2±22 (62)	0.78
6-C	22.4	25062±59 (47)	31829±68 (37)	90.4±27 (80)	0.79
7-B	25.1	9845±44 (35)	12400±50 (28)	93.1±26 (75)	0.79
7-C	25.1	13074±43 (33)	16404±50 (28)	102±23 (61)	0.80
7-E	23.5	146±5.0 (6.2)	191±5.7 (4.8)	71.9±21 (62)	0.77
7-G	20.8	245±7.5 (8.7)	291±8.2 (7.3)	192±29 (59)	0.84
8-E	16.8	20824±57 (45)	26345±65 (36)	108±25 (70)	0.79
8-F	16.8	33590±70 (55)	42576±81 (44)	110±28 (80)	0.79
8-H	14.9	2470±18 (15)	3162±21 (13)	101±18 (43)	0.78

a half-life.

b error shows one sigma standard deviation from counting statistics.

c detection limit.

For the evaluation of internal radiation exposure, three samples of mushrooms (*Trametes versicolor*) were collected at Kawauchi Village on December 20, 2011.

**Table 4:** Distribution of detected artificial radionuclides and ^40^K, ^134^Cs: ^137^Cs ratios and ^137^Cs: ^40^K ratios in mushrooms collected at Kawauchi Village, Fukushima Prefecture.

Points	Distance fromFNPP (km)	Radionuclide concentration in Bq/kg-dry			Radionuclide ratio	
		^134^Cs (2.1 y)^a^	^137^Cs (30 y)	^40^K (1.3×10^9^ y)	^134^Cs/^137^Cs	^137^Cs/^40^K
1–D	25.5	30196±28^b^ (23)^c^	38408±33 (19)	116±13 (35)	0.79	333
1–E	22.5	1596±5.4 (4.5)	2021±6.0 (3.7)	129±7.7 (16)	0.79	15.7
1–F	22.5	1630±7.1 (6.3)	2068±8.1 (4.8)	60±10 (29)	0.79	34.6

a half-life.

b error shows one sigma standard deviation from counting statistics.

c detection limit.

**Table 5:** Internal effective doses from mushrooms due to radiocesium and radiation doses by a gamma-ray survey meter in Kawauchi Village, Fukushima Prefecture.

Points	Distance from FNPP (km)	Internal effective dose in mSv/y	Air dose rate in µSv/h
1–D	25.5	7.0	0.63
1–E	22.5	0.37	0.46
1–F	22.5	0.38	0.46

The mass of soil samples collected in each area ranged from 20 to 134 g. The mass of tree needles ranged from 31 to 84 g and the mass of mushrooms ranged from 33 to 40 g. After collection, all samples were dried in a fixed temperature dryer (105°C, 24 h); then, soil samples were sieved for pebbles and organic materials (>2 mm) and tree needles and mushroom samples were broken into pieces before measurement of radionuclide activity.

After preparation, samples were put in plastic containers made of polypropylene and analyzed with a high purity germanium detector (ORTEC®, GEM35, Ortec International Inc., Oak Ridge, TN, USA) coupled to a multi-channel analyzer (MCA7600, Seiko EG&G Co., Ltd., Chiba, Japan) for 7,200–72,000 s. We set the measuring time to detect objective radionuclide levels. Gamma-ray peaks used for measurements were 604.66 keV for ^134^Cs (2.1 y), 661.64 keV for ^137^Cs, and 1460.75 keV for ^40^K (1.3×10^9^ y). Decay corrections were made based on sampling data. Detector efficiency calibration for different measurement geometries was performed using mixed activity standard volume sources (Japan Radioisotope Association, Tokyo, Japan). The relative detection efficiency of this instrument was 37.7%. Sample collection, processing, and analysis were executed in accordance with standard methods of radioactivity measurement authorized by the Ministry of Education, Culture, Sports, Science, and Technology, Japan (MEXT) [7]. All measurements were performed at Nagasaki Prefectural Institute for Environmental Research and Public Health, Nagasaki, Japan.

### Effective Dose

After measurements, external effective doses from soil samples were estimated from artificial radionuclide concentrations with the following formula:
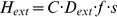
(1)in which *C* is the activity concentration of detected artificial radionuclides (radiocesium) [kBq/m^2^; estimated from the radiocesium concentration in Bq/kg-dry including soil particles (<2 mm) and collected areas of surface soil (0.00182 m^2^)]; *D_ext_* is the dose conversion coefficient reported as the kerma-rate in air 1 m above the ground per unit activity per unit area [(µGy/h)/(kBq/m^2^)], supposing that the kerma-rate in the air and the absorbed dose rate in the air are the same value, for radiocesium with the relaxation mass per unit area (β: g/cm^2^) set to 2.0 due to the passage of less than 1 year after the accident at FNPP [3.7×10^−3^ (µGy/h)/(kBq/m^2^) for ^134^Cs and 1.4×10^−3^ (µGy/h)/(kBq/m^2^) for ^137^Cs, ICRU 1994] [8]; *f* is the unit conversion coefficient (0.7 Sv/Gy for effective dose rate in the body per unit absorbed dose rate in air) [9], and *s* is the occupancy-shielding factor (0.36 for the public; 0.2 fractional time outdoors +0.8 fractional time indoors × 0.2 building shielding) [9] or the decrease in the coefficient by a shielding factor against exposure with gamma rays from a deposit 1 m above the ground (0.7 under the condition of usual land) [10]. Also, the annual external effective doses due to radioactive cesium (^134^Cs and ^137^Cs) were calculated using Eq. (1) (*H_ext_*×24 h/day×365 days).

Internal effective doses from mushrooms were estimated from radiocesium concentrations with the following formula:
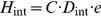
(2)where *C* is the activity concentration of detected artificial radionuclides (radiocesium) (Bq/kg-dry), *D_int_* is the dose conversion coefficient for adult intake (1.9×10^−5^ mSv/Bq for ^134^Cs and 1.3×10^−5^ mSv/Bq for ^137^Cs, ICRP 1996 [11]), and *e* is the estimated value of annual intake from the latest statistical data issued by the Ministry of Health, Labour, and Welfare, Japan in 2010 [12]. From this report, annual intakes of mushrooms were estimated at 6.5 kg/y in Japan, which is based on mean values of adults (>20 y).

## Results

Location and sample collection points in Kawauchi Village are shown in **Figure 1** and the distribution of detected artificial radionuclides in surface soil samples from Kawauchi Village is shown in **Table 1**. The prevalent dose-forming artificial radionuclides from all samples were ^134^Cs and ^137^Cs. Concentrations of radiocesium exceeded 8,000 Bq/kg-dry, the standard value for storing decontamination waste by Japanese guidelines, in some areas (1-A, 4-A, 5-A, 6-A, 7-A, 7-F, and 7-H) within the 20- to 30-km radius from FNPP (**Table 1**) [13]. These concentrations also exceeded 8,000 Bq/kg in areas within the 20-km radius from FNPP (8-A, 8-B, 8-C, 8-D, and 8-G). Radiocesium ratios (^134^Cs/^137^Cs) in soil samples other than 4-A ranged from 0.73 to 0.83 at the time of sampling.

The ^137^Cs deposition in these soil samples within a 20- to 30-km radius from FNPP ranged from 1.0±0.1 to 347.1±1.2 kBq/m^2^, whereas the ^137^Cs deposition in these samples within a 20-km radius from FNPP ranged from 376.7±1.3 to 6441.1±4.3 kBq/m^2^ (data not shown). For comparison, the ^137^Cs deposition was highest within a 30-km radius area surrounding the nuclear reactor of CNPP, known as the 30-km zone, and deposition densities exceeded 1,480 kBq/m^2^ in this zone [9].

The external effective doses from surface soil samples due to radiocesium and air dose rates in sampling places at Kawauchi Village are summarized in **Table 2**. The estimated external effective doses were 0.42–7.2 µSv/h (3.7–63.0 mSv/y) and air dose rates were over 2.3 µSv/h when soil samples were collected in areas within the 20-km radius from FNPP, whereas the estimated external effective doses were 0.0011–0.38 µSv/h (0.010–3.3 mSv/y) and air dose rates were 0.18–1.8 µSv/h when soil samples were collected in areas within the 20- to 30-km radius from FNPP.

Effective doses from soil samples and air dose rates in Kawauchi Village showed a significant positive relationship (r = 0.80, **Figure 2**). Moreover, as shown in **Figure 3**, effective doses were more than 0.42 µSv/h within the 20-km radius from FNPP, whereas they were below 0.38 µSv/h within the 20- to 30-km radius from FNPP.

The distribution of detected artificial radionuclides in tree needles from Kawauchi Village is shown in **Table 3**. Some concentrations of radiocesium exceeded 8,000 Bq/kg-dry (3-J, 6-C, 7-B, 7-C, 8-E, and 8-F). The radionuclide ratio of ^134^Cs to ^137^Cs (^134^Cs/^137^Cs) in tree needles ranged from 0.77 to 0.84, which was similar to results in soil samples.

Furthermore, the distribution of detected artificial radionuclides in mushrooms at Kawauchi Village is shown in **Table 4**. This mushroom (*Trametes versicolor*) is primarily used in medical supplies. Levels of radiocesium were extremely high in 1-D, although all concentrations of radiocesium exceeded 100 Bq/kg-dry, the new standard limit for general foods by Japanese guidelines [14]. Radionuclide ratios of ^137^Cs/^40^K ranged from 15.7 to 333. The internal effective doses from mushrooms due to radiocesium and air dose rates in sampling places at Kawauchi Village are summarized in **Table 5**. The estimated internal effective doses were 0.37–7.0 mSv/y and air dose rates were 0.46–0.63 µSv/h.

## Discussion

Kawauchi Village is located southwest of FNPP and included “a no-entry zone” (access-restricted area; revised on April 1, 2012) and “emergency evacuation preparation zones” that have been cancelled [2]. According to the interim report of the Investigation Committee on the Accidents at Fukushima Nuclear Power Stations of Tokyo Electric Power Company, there were 2,900 evacuees of Kawauchi Village: 400 residents of the “access restricted area” and 2,500 residents of “areas previously designated as the emergency evacuation preparation zones” (as of November 4, 2011) [15].

In the present study, the only detected artificial radionuclides in all samples from Kawauchi Village were ^134^Cs and ^137^Cs, and the ^134^Cs/^137^Cs values of these samples were around 0.80. Immediately after the accident, the ^134^Cs/^137^Cs values in areas to the south and southwest of FNPP were reportedly 0.9, which was higher than that observed around Chernobyl and similar to our results [4], [16]. Thus, it is suggested that the detected radiocesium from samples in the present study was derived from the FNPP accident. Moreover, the present study suggested that this environmental radioactivity was mainly derived from soil and fallen leaves existing in the surface of the earth because there was a strong relationship between air dose rates and estimated effective doses from soil samples. For example, the distribution patterns of ^134^Cs and ^137^Cs concentrations from pine and cedar tree needles were similar to those in the soil.

Our previous report showed that the prevalent dose-forming artificial radionuclides from soil samples of areas located at least 39 km away from FNPP were ^131^I,^ 134^Cs, ^137^Cs, ^129m^Te, ^95^Nb, and ^136^Cs immediately after the FNPP accident and that the prevalent dose-forming artificial radionuclides from these samples around FNPP were ^134^Cs, ^137^Cs, and ^129m^Te several months after the FNPP accident [17]. Therefore, detected artificial radionuclides around FNPP shifted primarily to radiocesium over several months [17]. This tendency is consistent with monitoring information of environmental radioactivity levels gathered by MEXT [18]. In addition, our previous report showed that estimated external effective doses from artificial radionuclide contamination in soil samples around FNPP were 1.9–2.9 µSv/h (8.7–17.8 mSv/y) in Fukushima City on March 22, 2011 [13]. By June 29, 2011, these estimated external effective doses were 0.25–0.88 µSv/h (2.2–7.6 mSv/y) [17]. In soil samples collected after the FNPP accident in early March, the highest soil depositions were measured to the northwest of FNPP, where the external dose rate 3 months after deposition was 4.8–98 µSv/h and the cumulative dose for 1 y was 51 to 1.0×10^3^ mSv; the highest values were at Futaba Yamada, 4.4 km away of FNPP, in highly contaminated regions near Futaba town [3]. At other locations from west to south of FNPP including Kawauchi Village, which are at least 22 km away from FNPP, the external dose rate at 3 months was 0.03–3.8 µSv/h and the cumulative dose for 1 y was 3–40 mSv [3].

National efforts are ongoing for reducing the estimated annual exposure dose to less than 20 mSv/y in the areas with an estimated annual exposure dose greater than 20 mSv/y (during the radiation emergency), and to bring the estimated annual exposure dose closer to 1 mSv/y in the areas with an estimated annual exposure dose of less than 20 mSv/y; this is being done with local authorities and inhabitants through the implementation of effective decontamination work (after the termination of the radiation emergency), in accordance with the recommendations of the International Commission on Radiological Protection (ICRP) [19]. The government especially aims to reduce the estimated annual exposure dose to 1 mSv/y as early as possible and continue with further reductions in children’s living spaces (such as schools or parks). The current situation after the FNPP accident corresponds to a radiological emergency and post-accident rehabilitation.

In the present study, the estimated external effective doses were 0.42–7.2 µSv/h (3.7–63.0 mSv/y) in areas within the 20-km radius from FNPP; the highest value was at 8-A, 16.8 km of FNPP. The estimated external effective doses were 0.0011–0.38 µSv/h (0.010–3.3 mSv/y) in areas within the 20- to 30-km radius from FNPP; the highest value was at 6-A, 23.0 km from FNPP. On the other hand, the air dose rate in the 20-km zone from FNPP was still high; the highest value was 65.1 µSv/h on December 20, 2011. Current levels of environmental contamination in this zone are almost the same [18]. It is especially clear that estimated effective doses were extremely low in areas within the 20- to 30-km radius from FNPP. Estimated effective doses in the present study are consistent with the estimated radiation dose reported by MEXT on December 11, 2011 [18]. Although a year has passed since the FNPP accident and levels of external exposure are certainly decreasing with the decay of artificial radionuclides, the remediation of contaminated soil due to artificial radionuclides may be effective as the result of crucial social responsibility in Japan and internationally. According to current results, the inhabitants of Kawauchi Village in areas within the 20- to 30-km radius from FNPP may return from evacuation areas because levels of external effective doses due to artificial radionuclides are sufficiently decreasing. Actually, the current levels of environmental contamination in Kawauchi Village are almost the same as the results of the present study [18]. Agricultural activities are being carefully carried out based on the guideline by the Nuclear Emergency Response Headquarters of the Japanese Government, with ongoing decontamination of farmlands and radiation monitoring [20]. Also, there is no restriction on agricultural activities within the 20- to 30-km radius from FNPP. Whether agricultural products can be shipped is determined based on the regulation values as a countermeasure. Similarly, the decontamination of forests is being carried out by suitable methods. In addition, thorough decontamination work is continuing in Kawauchi Village so that residents can safely live in their homes again. As a priority, the local authority of Kawauchi Village is working to facilitate the residents’ return to their homes, confirming low levels of environmental radioactivity.

One year after the FNPP accident, parts of the designated no-entry zones within the 20-km radius from FNPP were canceled and redesignated. Efforts should, however, be aimed at reducing any exposures that are above the reference level to a level that is below, if possible [19]. It is extremely important for residents to monitor artificial radionuclides in the environment and to dispose of contaminated soil due to artificial radionuclides as careful countermeasures.

More than 4,000 thyroid cancer cases were diagnosed during 1986–2002 among those who were children or adolescents (0–17 y) at the time of the Chernobyl accident in Belarus, Ukraine, and the four most contaminated regions of Russia [21]. Most of the thyroid dose was caused by the intake of ^131^I with food and cow’s milk during the first several weeks after the Chernobyl accident. Moreover, in the fungi soil system, cesium is considered a potassium (including ^40^K; 0.01% of all K) analog and is expected to behave like potassium because the elements are chemically similar and potassium availability affects the uptake and distribution of ^137^Cs [22]. In cases exceeding 1.0, the ^137^Cs/^40^K ratios could be an index of selective uptake of ^137^Cs. In general, mushrooms harvested from forests are known to be accumulators of ^137^Cs. In the present study, we confirmed that mushrooms selectively took in radiocesium instead of potassium. As a result, concentrations of radiocesium in mushrooms exceeded the new standard limit for general foods by Japanese guidelines and a part of internal effective doses also exceeded 1 mSv/y. Thus, careful attention is needed for consuming foods harvested from forests in order to avoid unnecessary chronic and low-dose exposure due to radiocesium, although current internal exposure, especially due to ^131^I through the consumption of locally produced food around FNPP, may be extremely small due to countermeasures taken from the initial phase of the accident.

There are several limitations in the present study. Radionuclides in soil samples may be unequally distributed around FNPP because the number of sampling points was relatively small and some areas had been already decontaminated, whereas other areas had not been decontaminated. In our study, there were gaps between air dose rates and estimated external effective doses. The bulk density of soils varies with their properties. Even if almost identical air dose rates or external effective doses were measured at sampling points, radioactivity in soil samples would vary with soil properties. In other words, air dose rates and external effective doses are not always proportional to radionuclide concentrations and/or radionuclide depositions, although our study showed a positive relationship between effective doses from soil samples and air dose rates (**Figure 2**). Although external effective doses from soil samples were estimated from radiocesium concentrations (kBq/m^2^; estimated from radionuclide concentrations in Bq/kg and collected areas of surface soil) after measurements of radionuclides, the weight of soil particles (<2 mm) mainly contributed to the activity concentration of radiocesium in kBq/m^2^. Moreover, concentrations of ^40^K showed that the nature of soils may also be related to the activity concentration of radiocesium. Therefore, radionuclide concentrations in Bq/kg are not always proportional to radionuclide concentrations in kBq/m^2^ (radionuclide depositions) and the radioactivity in soils and external effective doses from soils vary with soil properties. Further investigation with detailed conditions about external and internal effective doses is needed.

In conclusion, we evaluated current environmental contamination and contributions from external and internal exposures due to radiocesium in Kawauchi Village near FNPP and confirmed that current levels are sufficiently decreasing, especially in areas within 20- to 30-km radius from FNPP, although a certain amount of radiocesium derived from the accident was detected in samples from soil, tree needles, and mushrooms in this area. Inhabitants may return their homes with long-term follow-up of environmental monitoring and countermeasures such as decontamination and restrictions of the intake of foods for reducing unnecessary radiation exposure. The case of Kawauchi Village will be the first model for the return to residents’ homes after the FNPP accident.
